# Percutaneous coronary intervention assisted by invasive mechanical ventilation and intra-aortic balloon pump for acute myocardial infarction with cardiogenic shock: Retrospective cohort study and meta-analyses

**DOI:** 10.17305/bjbms.2019.4500

**Published:** 2020-11

**Authors:** Yin Liu, Chang-Ping Li, Peng-Ju Lu, Xu-Ying Wang, Jian-Yong Xiao, Ming-Dong Gao, Ji-Xiang Wang, Xiao-Wei Li, Nan Zhang, Chun-Jie Li, Jun Ma, Jing Gao

**Affiliations:** 1 Department of Cardiology, Tianjin Chest Hospital, Jinnan District, Tianjin, China; 2 Tianjin Medical University, Heping District, Tianjin, China; 3 Department of Prevention, Tianjin Children’s Hospital, Beichen District, Tianjin, China; 4 Cardiovascular Institute, Tianjin Chest Hospital, Jinnan District, Tianjin, China

**Keywords:** Invasive mechanical ventilation, IMV, intra-aortic balloon pump, IABP, percutaneous coronary intervention, PCI, mechanical circulatory support, acute myocardial infarction, cardiogenic shock

## Abstract

There is little evidence to recommend the optimal invasive mechanical ventilation (IMV) modes and ideal positive end-expiratory pressure stress levels for acute myocardial infarction-cardiogenic shock (AMI-CS) patients. The aim of this study was to compare the mortality outcome in patients with AMI-CS who were treated with percutaneous coronary intervention (PCI) assisted by intra-aortic balloon pump (IABP) + IMV with historical controls. From January 1, 2016 to June 1, 2017, 60 patients were retrospectively enrolled at Tianjin Chest Hospital. Out of these, 88.3% of patients achieved thrombolysis in myocardial infarction (TIMI) flow 3 after PCI. The all-cause mortality rate in-hospital and at 1 year was 25% (95% CI: 0.14–0.36) and 33.9% (0.22–0.46), respectively. A systematic review followed by meta-analysis was performed with four historical studies of patients treated by PCI + IMV with partial IABP, which found an in-hospital mortality rate of 66.0% (95% CI: 0.62–0.71). Recently, a meta-analysis of patients receiving PCI + IABP with partial IMV showed that the 1 year mortality rate was 52.2% (95% CI: 0.47–0.58). In Cox regression analysis of patient data from the current study, lactic acid level ≥4.5 mmol/L, hyperuricemia, and TIMI flow <3 were independent predictors of death at 1 year. All-cause mortality, in-hospital and at 1 year, in patients with AMI-CS treated with PCI + IABP and IMV was lower than in those treated with PCI + partial IABP or IMV. Larger, longer-term direct comparisons are warranted.

## INTRODUCTION

Cardiogenic shock (CS) in the setting of acute myocardial infarction (AMI) has increased in incidence to 10–12%, with a rate of associated in-hospital mortality as high as 30–50%. The in-hospital mortality is mostly secondary to hemodynamic deterioration, multiple organ failure, and systemic inflammatory response syndrome despite early revascularization treatment by the percutaneous coronary intervention (PCI) or coronary artery bypass grafting (CABG) [1-5].

Treatment of AMI-CS often requires multimodal treatment strategies which remain to be clearly defined [1]. The treatment strategies can include intra-aortic balloon pump (IABP), which is the most widely used mechanical circulatory support (MCS) modality in patients with AMI-CS; however, in the IABP-SHOCK II trial it did not significantly reduce mortality rates within 1 year follow-up period [5,6], and the current guidelines do not recommend its routine use (III/B evidence) except for cases with mechanical complications or hemodynamic instability (IIa/C evidence) [7]. The extracorporeal membrane oxygenation (ECMO), Impella, TandemHeart, and subcutaneous MCS devices may reduce the mortality rate, but further studies are warranted [8-10]. Patients with AMI-CS also often require invasive mechanical ventilation (IMV) to improve respiratory function, correct hypoxemia, and stabilize hemodynamics [1,11]. However, as indicated in the 2017 statement on CS management by the American Heart Association, there is little evidence to recommend the optimal IMV modes and ideal positive end-expiratory pressure (PEEP) stress levels for AMI-CS patients [1].

In 2015, our medical center established a multidisciplinary CS treatment team that mainly uses a combination therapeutic strategy, namely, early revascularization therapy with PCI assisted by IABP plus IMV (PCI + IABP + IMV), in patients with AMI-CS and refractory hypoxemia; its effect on improving the mortality rate is the subject of this study.

## MATERIALS AND METHODS

### Overall study design

**FIGURE 1:**
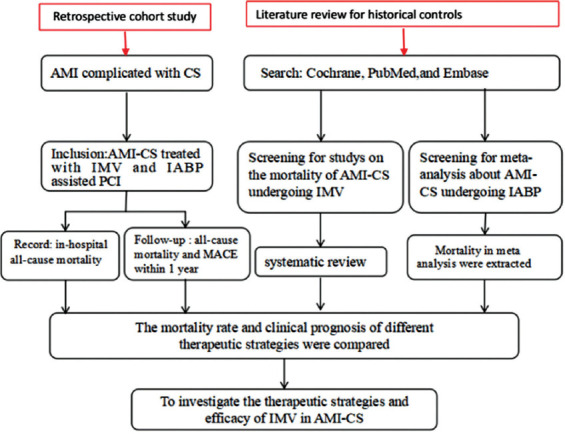
Overall study design. AMI: Acute myocardial infarction; CS: Cardiogenic shock; IMV: Invasive mechanical ventilation; IABP: Intra-aortic balloon pump; PCI: Percutaneous coronary intervention; MACE: Major adverse cardiac events.

The overall study design is shown in Figure 1. This study consisted of three parts: a retrospective cohort study, a self-performed meta-analysis of AMI-CS patients treated with PCI assisted by IMV disregarding IABP usage (PCI + IMV + partial IABP) as historical control, and a systematic review of published meta-analyses of AMI-CS patients treated with PCI assisted by IABP disregarding IMV usage (PCI + IABP + partial IMV), also serving as historical control.

### Retrospective cohort study of AMI-CS patients treated with PCI + IABP + IMV

This retrospective cohort study enrolled patients with AMI-CS who were admitted to Tianjin Chest Hospital from January 1, 2016 to June 1, 2017 and treated with PCI + IABP + IMV. Our medical center is the core hospital of a first-tier city in China (population, 15.4 million) and provides AMI medical services (>1500 annual emergency PCI caseload). The study inclusion criteria were: (1) age >18 years and (2) diagnosed with both AMI and CS, with the latter defined as one or more of the following: systolic blood pressure <90 mmHg for 30 minutes or a need for vasoactive drugs to maintain systolic blood pressure at ≥90 mmHg, pulmonary congestion, and insufficient perfusion of at least one organ as manifested by altered mental status, clammy skin, urine volume <30 mL/h, or blood lactate level >2.0 mmol/L. The exclusion criteria were: (1) shock caused by mechanical complications of AMI, such as rupture of ventricular septum, free wall, papillary muscles or others; (2) shock other than CS, such as septic or hypovolemic shock; (3) CABG; and (4) other MCS.

The current retrospective study was approved by the Ethics Committee of Tianjin Chest Hospital and complied with the 1975 Declaration of Helsinki. Patients provided written informed consent.

Trial registration: the study is registered on ClinicalTrials.gov (NCT03600259).

### Meta-analysis of AMI-CS patients treated with PCI + IMV + partial IABP

For patients treated with PCI + IMV + partial IABP (a part of patients with AMI combined with CS received IABP treatment), the PubMed, Embase, and Central databases were searched for possible relevant studies published from January 1997 to December 2017 using the following index keywords: “myocardial infarction”, “cardiogenic shock”, “mechanical ventilation”, “invasive mechanical ventilation”, or “respiration, artificial”. The inclusion criteria were: (1) study type: ­observational study or clinical trial; (2) study subject: AMI patients with CS; (3) interventions: IMV therapy, with other treatment strategies including one or more of the following: reperfusion therapy (PCI, CABG, or thrombolysis treatment), mechanical circulation support (IABP or other), drug treatment or others; (4) outcomes: the main outcome was in-hospital all-cause mortality rate, and the secondary outcomes were 30-day, 6-month, and 1-year all-cause mortality rates; and (5) studies published in English. Studies using non-invasive ventilation and those that did not clearly define invasive or non-invasive ventilation were excluded from the study. Studies not reporting the number of deaths or mortality rates of patients with mechanical ventilation were also excluded from the study. Two researchers used the same data acquisition table to independently collect data. In cases of disagreement, a third-party member was involved. Stata Statistical Software: Release 12 (STATA 12.0, StataCorp LP., College Station, TX) was used for meta-analysis. A fixed-effects model was adopted according to the heterogeneity test, given an I^2^ value of <50% and *p* < 0.05. A pooled effect was derived as mortality rate with 95% confidence intervals (95% CI) from the meta-analysis.

### Systematic review of published meta-analyses of AMI-CS patients treated with PCI + IABP + partial IMV

For patients with AMI-CS treated with PCI + IABP + partial IMV (a part of patients with AMI combined with CS received IMV treatment), the keywords “intra-aortic balloon pump”, “myocardial infarction”, and “cardiogenic shock” were used to retrieve from the Cochrane, PubMed, and Embase database the latest published meta-analyses. Five studies were considered, including the study by Unverzagt et al. that included seven randomized controlled trials [12]. The Unverzagt study retrieved from the Cochrane was ultimately used in this study because the other four meta-analyses published after 2015 included case reports and had less rigorously defined outcomes [13-16].

### Data collection and follow-up

Demographic characteristics, medical history, laboratory test results, interventional and other treatment data, and patient complications were collected from electronic medical records. Left ventricular ejection fraction (LVEF) was measured by echocardiography before treatment. Hemodynamic parameters were derived from the first Swan-Ganz catheter measurement after PCI. The peak levels of high-sensitivity cardiac troponin I (hs-cTNI) and N-terminal pro b-type natriuretic peptide (NT-proBNP) were measured by biochemical analysis. Glomerular filtration rate was estimated using the Modification of Diet in Renal Disease method. Other ­l­aboratory biochemical parameters were assessed at baseline, which was before the initiation of treatment post-hospital admission.

Case inquiries and follow-up through visits or telephone were performed during hospitalization, and at 1, 3, 6, and 12 months after AMI onset. All-cause death and major adverse cardiac events [MACE] (cardiac death, recurrent myocardial infarction, target vessel revascularization, and hospitalization for heart failure and stroke) were recorded.

### Treatment strategy

The indications for IMV were an inability to normalize blood oxygen after room air, i.e., SaO_2_ <90% or PO_2_ <60 mmHg, PCO_2_ >50 mmHg, and pH <7.35. Patients received general anesthesia in addition to tracheal intubation and HAMILTON-C1 ventilation. The protective lung ventilation strategy was used, including synchronized intermittent mandatory ventilation, pressure support ventilation (PSV), and PEEP modes. The initial tidal volume in the PSV mode was set to 4–8 ml/kg, and pressure to <30 cm H_2_O. PEEP pressure was maintained at 5–10 cm H_2_O. Ventilator parameter settings were adjusted according to patient’s status. After passing the spontaneous breathing test for 30 minutes, IMV support was discontinued as per the standard established by the European Respiratory Society in 2007 [17].

For MCS, our medical center has IABP and ECMO devices, which are the only ones commercially available in China. In this study, the IABP device MAQUETCS100 (USA) was used and indicated for patients in whom hemodynamic stability could not be restored after active drug therapy. Left or right femoral artery puncture was initiated prior to PCI for IABP catheter (MAQUET Co., USA) placement. In electrocardiography, R wave with 1:1 mode was triggered. The criteria for IABP discontinuation were one or more of the following: stable hemodynamics with low dose or no vasoactive drugs, urine output >1 ml·kg^-1^·h^-1^, mean arterial pressure >70 mmHg, and pulmonary capillary wedge pressure <18 mmHg.

For PCI, blood oxygen saturation was maintained at >90%. Aspirin 300 mg and ticagrelor 180 mg were administered through nasogastric tube. Revascularization of the infarct-related artery (IRA) was performed after completing coronary angiography through femoral artery access. Concomitant revascularization of non-IRA was at the operator’s discretion. Two experienced cardiologists decided treatment strategy for each patient. All patients were admitted to the coronary intensive care unit after PCI and were treated according to guidelines [18-20].

### Outcomes

The primary study outcome was in-hospital all-cause mortality rate, while secondary outcomes were 30 days, 6 months, and 1 year mortality. During the 1-year follow-up, the incidence of MACE defined as cardiogenic death, recurrent myocardial infarction (per global unified definition, i.e., after 28 days of index AMI [21]), target vessel revascularization (of any part of the target vessel, by PCI or CABG), and hospitalization for heart failure (diagnosed per 2016 European Heart Association guidelines [22]), or stroke (cerebral ischemia or hemorrhage by computed tomography and/or magnetic resonance imaging) was recorded. This study also evaluated hospitalization duration and rate of in-hospital ventilator-associated pneumonia (diagnosed according to the 2005 American Thoracic Society guidelines [23]).

### Statistical analysis

SAS 9.3 statistical software (SAS Institute Inc., Cary, NC, USA) was used for statistical analyses. After analyzing the distribution pattern of continuous variables, mean with standard deviation (SD) was used for normally distributed data; otherwise, median with interquartile range (IQR) was used. Categorical data are presented as number of patients (%). Cox proportional hazards regression model was used for multivariable analysis of the risk factors of death at 1 year. A *p* value of <0.05 was considered statistically significant. Log-rank test was used to analyze survival curves.

## RESULTS

### Retrospective cohort study of AMI-CS patients treated with PCI + IABP + IMV baseline characteristics and results of laboratory testing

Of 189 patients with AMI-CS screened upon retrospective chart and electronic records review, 129 were excluded (5 with mechanical complications; 2 with non-CS; 11 received CABG; 12 received ECMO; 18 received PCI with IMV alone; 35 received PCI with IABP alone; 21 received PCI only; and 25 did not undergo revascularization). Therefore, a total of 60 patients were included for analysis, and their baseline characteristics and laboratory results are shown in Table 1. The mean age of patients was 67 years, with 71.7% of total patients being men. Sixty-five percent of patients had AMI of ST-segment elevation. While all patients underwent primary PCI, 33.9% of those who had multivessel disease (19/56) received multivessel PCI additionally. Of the 60 patients, 49 patients had pre-hospitalization CS, 10 patients developed CS within 24 hours of admission, and 1 developed CS 24 hours after admission. All 60 patients (100%) underwent emergency PCI after AM-CS following admission, with 100% PCI success rate. Both IABP and IMV were initiated before PCI. The median treatment period of IABP-assisted PCI was 3 days (IQR, 2.0–5.0; overall range, 1.0–12.0), and the median treatment period of IMV-assisted PCI was 2 days (IQR, 1.0–3.0; overall range, 1.0–11.0). The PSV + PEEP mode was used in IMV. The pressure of PEEP mode was set to 5–10 cm H_2_O.

**TABLE 1:**
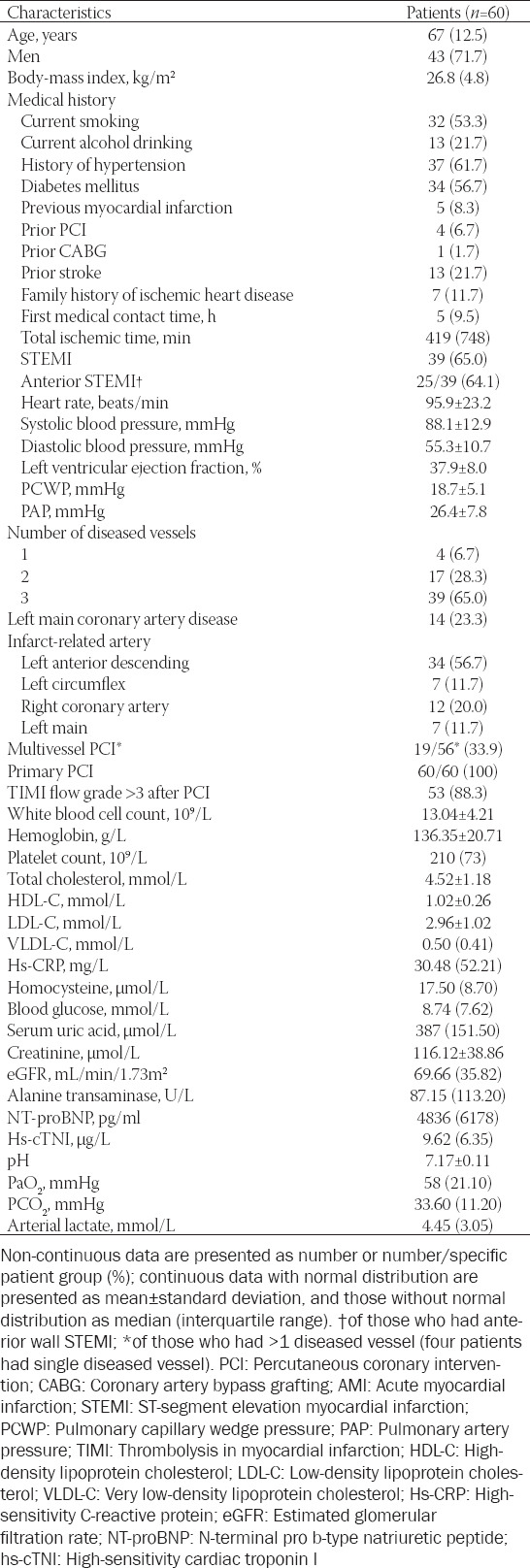
Baseline characteristics and laboratory parameters of patients included in the retrospective cohort study

### Cohort study outcomes

One patient was lost to follow-up before the 30-day follow-up. The remaining patients were followed up for 1 year, with a total of 20 deaths among them. The study outcomes are shown in Table 2. Fifteen deaths occurred in hospital from multiple organ failure (9 patients), sudden cardiac deaths (5 patients), and severe gastrointestinal bleeding (1 patient). During the 30-day follow-up, 2 patients died of severe heart failure, and 3 more patients died from severe heart failure during the remaining period of the 1-year follow-up. Therefore, the all-cause mortality rate in hospital, at 30 days, 6 months, and 1 year was 25.0% (95% CI: 0.14–0.36), 28.8% (0.17–0.41), 32.2% (0.20–0.44), and 33.9% (0.22–0.46), respectively.

**TABLE 2:**
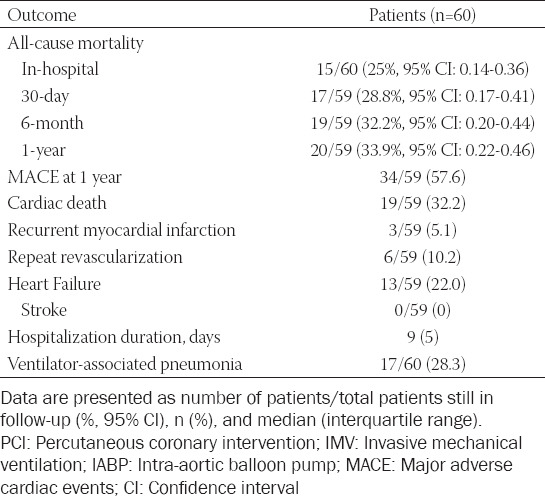
Clinical outcomes of patients treated with PCI assisted by both IMV and IABP (PCI+IMV+IABP) in the retrospective cohort study

### Meta-analysis of PCI + IMV + partial IABP in the treatment of patients with AMI-CS

**FIGURE 2:**
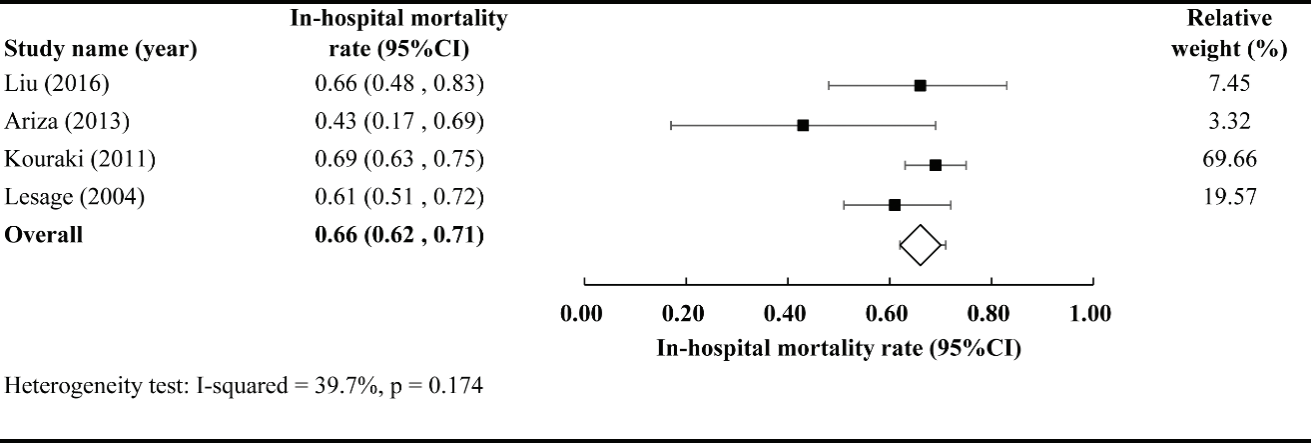
In-hospital mortality of patients with acute myocardial infarction-cardiogenic shock (AMI-CS) receiving percutaneous coronary intervention (PCI) assisted by invasive mechanical ventilation (IMV) and partial intra-aortic balloon pump [IABP] (PCI + IMV + partial IABP).

Of the 1317 records identified through the database search, 304 duplicates were removed and 965 were excluded because they were not written in English (n = 112), were neither human observational studies nor clinical trials (n = 358), or were ­unrelated to our research objectives (n = 499). Of the remaining 48 articles, 43 were excluded because of poorly defined IMV use or unreported mortality data. Therefore, five studies were selected for detailed review [24-28], and the study by Kontoyannis et al. [24] was further excluded due to the patient group being small (10 treated with IMV) and the lack of early emergency revascularization therapy by PCI. Finally, four studies in total were included for meta-analysis, and all data from patients treated with IMV were extracted [25-28]. All four studies reported in-hospital mortality rate, while only one study reported 28-day mortality rate [25]. None of the studies reported mortality rates at 30 days, 6 months, or 1 year. IABP and revascularization (PCI or CABG) were used occasionally in the treatment, in all four studies. Randomized effects models were used to combine the results. The in-hospital mortality rate of AMI-CS patients treated with PCI + IMV + partial IABP was 66% [95% CI: 0.62–0.71] (Figure 2).

### Historical data from published meta-analyses of PCI + IABP + partial IMV in the treatment of patients with AMI-CS

The meta-analysis by Unverzagt et al. from 2015 included seven randomized control studies with a total of 790 cases [12]. All patients were treated with IABP and revascularization, and some additionally with IMV. As a combined result with a fixed effects model, five studies reported in-hospital mortality rate of 36% (95% CI: 0.31–0.41); six studies reported 30-day mortality rate of 40.1% (95% CI: 0.35–0.45); four studies reported 6-month mortality rate of 48.7% (95% CI: 0.43–0.54), and two studies reported 1-year mortality rate of 52.2% (95% CI: 0.47–0.58).

### Comparison of mortality rate among the three therapeutic strategies

**FIGURE 3:**
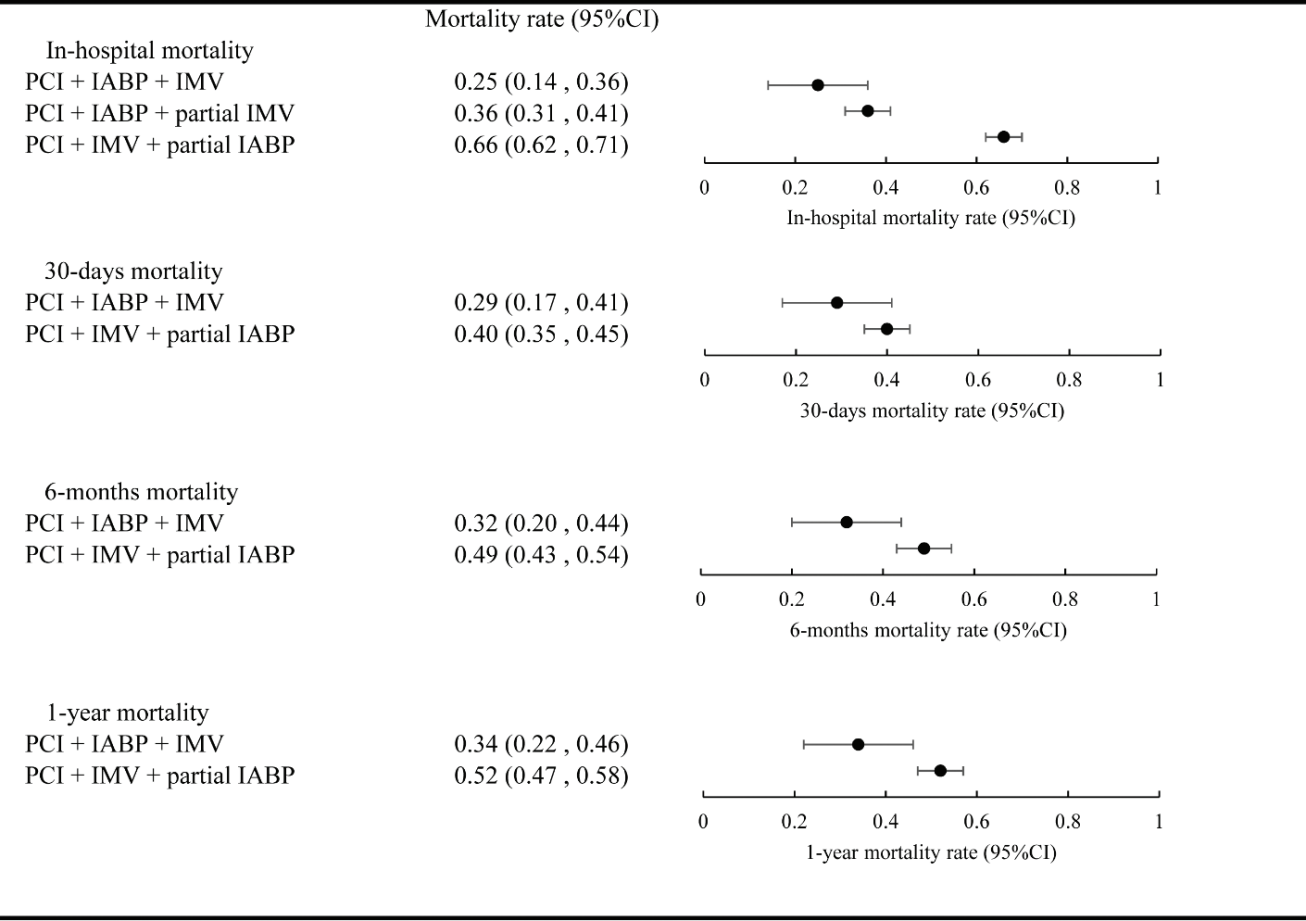
Comparisons of mortality among the three different therapeutic strategies at indicated time points. PCI: Percutaneous coronary intervention; IABP: Intra-aortic balloon pump; IMV: Invasive mechanical ventilation.

Comparing in-hospital, at 30 days, 6 months, and 1 year all-cause mortality rates of patients treated with PCI + IABP + IMV (Table 2) with those from the historical data for PCI + IMV + partial IABP from our meta-analysis and the published PCI + IABP + partial IMV meta-analysis (Figure 3), we found that the mortality rates of patients treated with PCI + IABP + IMV were lower than historical data at all time-points. In particular, the difference in in-hospital mortality rates between patients treated with PCI + IABP + IMV and those treated with PCI + IMV + partial IABP was the most evident (25% [95% CI: 0.14–0.36] vs. 66% [95% CI: 0.62–0.71]). Furthermore, the difference between patients treated with PCI + IABP + IMV and those treated with PCI + IABP + partial IMV gradually increased over time, and was most noticeable at 1 year (33.9% [95% CI: 0.22–0.46] vs. 52.2% [0.47–0.58]).

### Survival analysis

**FIGURE 4:**
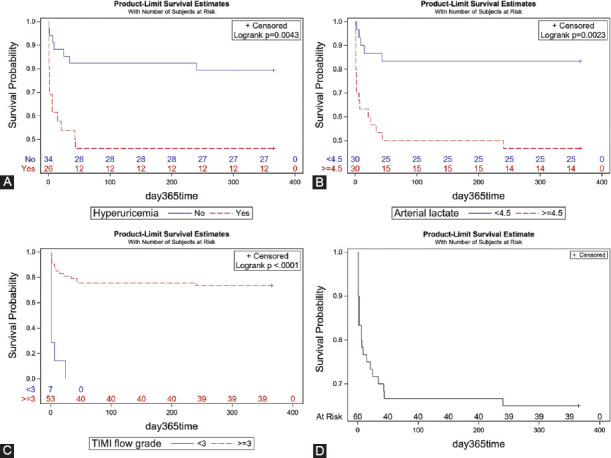
Time-to-event curves of subgroups segregated based on the independent risk factors for 1-year all-cause mortality. (A) Hyperuricemia, (B) arterial lactate, (C) thrombolysis in myocardial infarction (TIMI) flow grade, (D) all patients.

In multivariable regression analysis, lactic acid level ≥4.5 mmol/L (hazard ratio: 3.99 [95% CI: 1.29–12.34]), hyperuricemia (4.51 [1.63–12.45]), and thrombolysis in myocardial infarction (TIMI) flow <3 (12.1 [3.71–39.43]) were independent risk factors (all *p* ≤ 0.05) of death at 1 year (Table 3). Receiver operating characteristic curve analysis in conjunction with Youden’s index was used to determine the intercept point of the lactic acid level. The survival curves of subgroups segregated by these three independent risk factors, analyzed by the log-rank test, are shown in Figure 4.

**TABLE 3:**
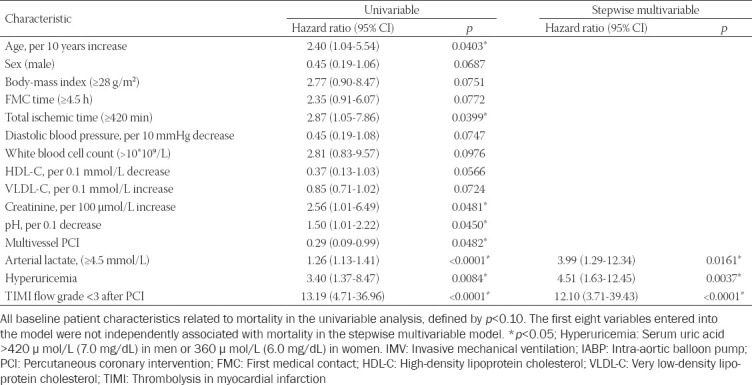
Predictors of 1-year mortality rate by univariable and stepwise multivariable Cox regression analyses after treatment with PCI+IMV+partial IABP

## DISCUSSION

Our medical center established a multidisciplinary team including cardiology, cardiac surgery, emergency department, coronary care unit, ultrasound, extracorporeal circulation, and anesthesia for the treatment of CS cases. In this study, we analyzed mortality outcomes in patients with AMI-CS in whom IMV and IABP were used to assist PCI treatment. In comparison with historical controls for PCI + IMV or PCI + IABP derived from systematic literature reviews, PCI + IABP + IMV statistically decreased 1-year mortality rate by 18%, which might be attributed to early revascularization along with improvement of hypoperfusion and hypoxemia correction.

Hayıroğlu et al. found that higher SYNTAX score II was associated with poor prognosis [29]. Considering the impact of coronary anatomy on prognosis, we calculated that the proportion of three-vessel lesions in the meta-analysis for IABP + PCI (315 people) was 52.1%, which was lower than that in our cohort study (65%). Although the coronary anatomy in our cohort population is more complex, we achieved a lower mortality through a combination of treatment strategies.

Early invasive revascularization strategies are key to reduce CS-associated mortality and are recommended by the American and European Heart associations [1,7]. Multivessel disease is common in patients with AMI-CS and, in the present study, 93.3% of patients had multivessel disease while 33.9% of patients were treated with multivessel PCI. In the CULPRIT-SHOCK randomized controlled trial, the composite endpoint of all-cause mortality and renal replacement therapy was higher at 30 days in the multivessel disease-treated group than in the IRA alone-treated group [5], while in a multicenter prospective cohort study of patients with AMI-CS, multivessel PCI significantly reduced all-cause mortality rate and recurrent risk of revascularization [30].

Patients with AMI-CS suffer from myocardial stunning even after revascularization. In another study, the follow-up of survivors discharged from hospitals documented a 22.4% 1-year mortality rate. Over 30% of patients exhibited various degrees of heart failure symptoms and eventually developed chronic heart failure, having a heavy burden on families and society [31].

In this study, at 1-year follow-up the incidence of chronic heart failure among patients with MACE was significantly reduced to 22.0%. Among them, 19 cases had complete revascularization with 2 cases of heart failure (15.8%), and 37 cases showed incomplete revascularization with 8 cases of heart failure (27%), which suggests that complete revascularization might be more beneficial to recovery from myocardial stunning. Larger studies with longer-term follow-up are warranted to inform optimal treatment strategy for effective circulation and respiratory assisted early revascularization therapy for patients with AMI-CS.

AMI with CS is often accompanied by respiratory distress, respiratory failure, and severe blood gas abnormalities, requiring mechanical ventilation support, which was used in around 80% of patients in the SHOCK, IABP-SHOCK II, and CULPRIT-SHOCK trials and in 100% in an Impella randomized controlled trial. However, there was no specific information to identify the ventilation approach and mode [4,5,9]. In the non-randomized controlled trial by Kontoyannis et al. of 18 cases of patients with AMI-CS [24], CMV + PEEP (pressure set to 10 cm H_2_O) synergized with IABP in hemodynamic stabilization with an in-hospital mortality rate of 20%. In contrast, in the study of Liu et al. from 2016 who retrospectively reviewed and analyzed 62 cases of STEMI patients with CS, PSV + PEEP (pressure set to 4–10 cm H_2_O) was used and did not reduce the mortality rate; the in-hospital mortality rate was 65.5% [25].

The protective lung ventilation strategy is a new concept; its purpose is to correct blood gas anomalies to minimize lung injury caused by mechanical ventilation [32]. PEEP mode helps gas exchange and lung recruitment, and the appropriate pressure setting can lower the burden on front and back sides of the left ventricle, reduce pulmonary edema, and pulmonary vasoconstriction caused by hypoxia, which ultimately reduce pulmonary resistance and increase cardiac index [33]. In this study, protective lung ventilation strategy was used along with IABP and PCI. Ventilators were set to lower tidal volume and pressure, and the low to medium pressure of PEEP mode was given simultaneously. However, the results were inconclusive in terms of mortality rate reduction, which was only numerical as compared to historical controls.

In the current study, tracheal intubation was used for mechanical ventilation. The median time was 2 days and the incidence of ventilator-associated pneumonia was 28.3%, which appears comparable to the ~20% reported for critically-ill patients receiving mechanical ventilation [34]. Tracheal intubation increases the risk for respiratory infections, and non-IMV should be considered in CS patients without severe hemodynamic instability [35].

Patients with AMI-CS have a high mortality rate, rendering it clinically relevant to define risk factors. In the present study, the 95% CI of the three risk factors identified was rather broad likely because of small sample size; however, the survival curves underscore the relevance of the factors identified. Two of the three independent risk factors, namely TIMI flow <3 and serum lactate level after surgery, had been reported [6,36,37]. Although the treatment was different, similar risk factors could be identified. Hayıroğlu et al. analyzed the risk factors for in-hospital mortality in 319 patients with CS [38]. The percentage of TIMI 3 flow after PCI (54.1%) and the insertion rate of IABP (26.7%) are lower than in our study, but the conclusion of the study is similar to ours. It also confirms that TIMI flow grade<3 after PCI (odds ratio [OR]: 2.57, 95% CI: 1.32–5.00) and lactate (OR: 1.76, 95% CI: 1.48–2.10) are the strongest risk factors. This suggests that the risk factors for death are related to the nature of CS. The third risk factor for death at 1 year identified in this study of patients with AMI-CS was hyperuricemia, which had been identified as an independent risk factor for AMI [39]; however, Liu et al. retrospectively analyzed 951 cases of STEMI patients and found that hyperuricemia increased the mortality rate of Killip I STEMI and was unrelated to the mortality of Killip II-IV STEMI [40].

This study has several limitations. First, the sample size was small and follow-up duration short. Based on the in-hospital mortality rates observed in this study, one could estimate that a minimum sample size of 100 cases should better assess statistical significance. Second, this was a single-center and non-randomized controlled trial. However, AMI patients with CS are usually in critical condition, rendering it challenging to perform multicenter, prospective, and randomized controlled clinical studies. Third, although comparisons with historical controls derived from systematic literature reviews and meta-analyses provided a framework for discussion of the results in this study, a comprehensive prognostic assessment is precluded due to lack of direct comparison of treatment complications and MACE events.

In this single-center study of patients with AMI-CS and refractory hypoxemia, early emergency PCI treatment assisted by IABP and a protective lung ventilation strategy for IMV with PEEP mode set to 5–10 cm H_2_O evidently reduced the in-hospital and 1-year mortality rates relative to those for historical controls for PCI assisted either by IMV or IABP, respectively. Larger sample size, long-term follow-up, and multicenter clinical trials are warranted to verify the efficacy and long-term prognosis of the approach.
